# Comparison of Residual Triple Antibiotic Paste, Propolis and Calcium Hydroxide on Root Canal Walls in Natural Open Apex Teeth: An *In Vitro* Study 

**DOI:** 10.22037/iej.v13i1.15807

**Published:** 2018

**Authors:** Armita Rouhani, Mahbobe Erfanzadeh, Hamid Jafarzadeh, Elham Najafi

**Affiliations:** a *Dental Research Center, Mashhad University of Medical Sciences, Mashhad, Iran; *; b *Private Practice, Mashhad, Iran; *; c *Department of Endodontics, Dental School, Qom University of Medical Sciences, Qom, Iran*

**Keywords:** Calcium Hydroxide, Intracanal Medicament, Open Apex, Propolis, Triple Antibiotic Paste

## Abstract

**Introduction:**

Endodontic therapy is challenging in open apex teeth. One of these problems is the residue of medicaments on root canal walls. The aim of this study was to evaluate the amount of residual materials on canal walls after the use as medicaments within natural open apex teeth.

**Methods and Materials:**

A total of 45 human extracted single-rooted premolars with open apices were selected. After cutting off the crowns, root canals were gently instrumented using #40 files and irrigated with 0.5% sodium hypochlorite. The samples were randomly divided into three groups: calcium hydroxide (CH), triple antibiotic paste (TAP) and propolis (PP). In these groups, CH, TAP, or PP were placed into the canals, respectively. The samples were then restored with temporary fillings. After one week, instrumentation was again performed as mentioned above. The samples were longitudinally cut and scanned and the remaining material in both halves was evaluated using computer software. One-way ANOVA was used to compare the average paste level remaining on the canal walls.

**Results:**

The residual amount of CH on the canal walls was significantly higher than that of PP (*P*=0.001). The residual amount of CH was higher than TAP but this difference was not significant (*P*=0.144); the residual amount of TAP was higher than PP but this difference was not significant, either (*P*=0.094).

**Conclusion:**

PP is superior to CH and TAP in terms of removability from the root canal system within open apex teeth.

## Introduction

Microorganisms and their by-products play a key role in the pathogenesis of apical periodontitis. Although the number of microorganisms is reduced as much as possible by mechanical and chemical methods during endodontic treatment, a number of irritants may remain due to the complex anatomy of the root canal system [1]. Therefore, the use of various medicaments within the root canal between therapy sessions is recommended for chemical cleansing. This is more important for immature teeth with open apices where mechanical instrumentation is less likely to be performed thoroughly, because the thin root canal walls do not allow this without severe damage to the tooth.

Calcium hydroxide (CH) is widely used as an intracanal medicament for disinfection and accelerating the repair process of periapical lesions. In general, intracanal medicaments are used within canal due to antimicrobial activity, neutralizing the tissue remnants within the canal as well as prevention and control of pain after treatment [2]. CH is widely used as an intracanal medicament [3].

Propolis (PP) is a brown resinous substance produced by honey bees. In the last 30 years, several studies suggest the anti-inflammatory effects of honey and PP. Galangin, one of the active components of propolis, is the most effective agent inhibiting COX activity, especially COX2 and lipoxygenase. Given the known properties of PP as well as its low cytotoxicity compared to CH, it may be an acceptable option as an intracanal medicament [4].

Triple antibiotic paste (TAP) contains ciprofloxacin, metronidazole and minocycline and has been introduced as an intracanal medicament, especially for disinfecting the root canal prior to regenerative endodontic treatment [5]. This triple paste is effective against the pathogens commonly found in root canals, including *Enterococcus faecalis* [6, 7]. In the new therapeutic protocol of revascularization for permanent necrotic teeth, the root canal is disinfected using sodium hypochlorite, and then the canal is filled with TAP. It has been shown that the combination of these three antibiotics can eliminate bacteria in the deepest layers of root canal dentin [8, 9].

Ding *et al.* [10] in 2009 concluded that after placing TAP in the canal and sealing the access cavity with MTA, complete development of roots was observed in radiographs of patients and the teeth responded positively to pulp testing. Thomson *et al.* [8] observed that after using TAP and MTA, apical periodontitis and sinus tracts of patients were improved during 18-month follow-up of the patients. The roots continued to develop and apical closure was observed.

Intracanal medicaments may remain on root canal walls what may cause problems. The residual material on canal walls adversely affects the strength of dentin bonding and also prevents the penetration of sealer into dentinal tubules [11, 12]. The aim of this study was to evaluate the amount of residual above-mentioned materials on canal walls after their use as intracanal medicaments in teeth with natural open apices.

## Materials and Methods

This study was experimentally conducted on 45 human extracted single-rooted premolars with open apices. The size of the apical foramen was set to be between #100 and #120 files to approximately equalize the samples. The samples were also evaluated for absence of cracks and fractures at the root surfaces. The crowns of all the teeth were cut off by a non-stop device using a coal disk (LM, Italy) to achieve the same length for all roots (12 mm). Working length was visually determined. This means that a #40 File (K-File, Sendoline, Sweden) was placed into the root canal so that its tip was visible at the apical foramen. The working length was determined by subtracting 1 mm from this length. At the end of each roots, a piece of cotton was placed to simulate periapical tissue and the samples were mounted in a putty molding material (Speedex, Asia Chemi Teb, Tehran, Tabriz, Iran).

For canal cleansing, gentle peripheral instrumentation was done at the canal walls using a #40 file and irrigation with 5 mL of 0.5% sodium hypochlorite. After irrigation, the canals were dried with paper cones (Orca; Tiagin, China). Then the samples were randomly divided into three groups: CH, TAP and PP. In the CH group, CH powder (Golchay, Tehran, Iran) was prepared in paste form and was placed into the canal using a #40 file. Ca paste was formed by mixing calcium hydroxide powder with distilled water with a powder to liquid ratio of 1:2.

In TAP group, triple antibiotic paste, a mixture of three antibiotics of ciprofloxacin, metronidazole and minocycline with 1:1:1 ratio in distilled water, was prepared with a powder to liquid ratio of 1:3 and placed into the canal using a #40 file. *Hoshino ratio* was used to prepare TAP. This ratio states that 200 mg ciprofloxacin with 500 mg metronidazole and 100 mg minocycline gives rise to 1: 1: 1 ratio [7]. 

In PP group, propolis, which was prepared in paste form, was placed into root canal. Due to proprietary production of PP, its formulation is not available. Each milliliter of the prepared water based PP contains 5 mg of the active ingredient of polyphenol. This compound was prepared in paste form in Mashhad Razi Research Institute.

After placing the desired material into the root canal, the coronal part of the respective tooth was sealed with coltosol (Aria Dent, Tehran, Iran) for one week. Then, the temporary filling was removed using a fissure bur (Tizkavan, Tehran, Iran) and instrumentation was done again using gentle filing with a #40 file and irrigation with 5 mL of 0.5% sodium hypochlorite.

**Table 1:** Mean (SD), minimum, maximum and result of one-way ANOVA

	**Mean (SD)**	**Min**	**Max**	***P-*** **value**
**CH (15)**	43.7 (19.69)	6.64	72.12	F=8.303 P=0.001
**TAP (15)**	30.37 (15.91)	14.24	66.34	
**PP (15)**	19.77 (7.)	6.34	35.04	

**Figure 1:**
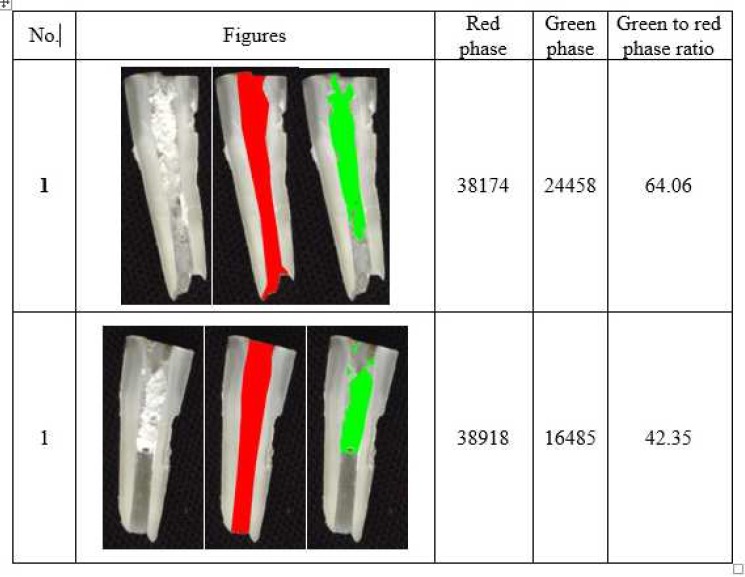
Example of calcium hydroxide paste residual in canal (*Red Phase*: Total area of root canal; *Green Phase*: Area of the medicament remaining within the canal)

The samples were fixed on acrylic plates and were longitudinally cut by a microcutting CNC device, so that each sample was divided into two parts. The samples were then scanned with a UMAX scanner (PowerLook 2100XL) and the amount of residual material in both halves of each sample was investigated using a microstructure image processing software (MIP version 4, Nahamin Pardazan Asia Company, Tehran, Iran). The total area of each tooth and the area where the medicament has remained within the root canal were calculated using MIP and their ratio was determined.

Because each tooth is divided into two parts, the ratio of the two parts were averaged to obtain the overall proportion. Normal distribution of data and the average levels of residual paste on the canal walls were evaluated using Shapiro-Wilk test and one-way ANOVA, respectively.

## Results

Two teeth containing PP were excluded from the study during experimental procedures, so 13 teeth were examined in the PP group.  The normality of data distribution was confirmed using

Shapiro-Wilk tests. One-way ANOVA showed that three types of paste were statistically significant from each other regarding residual medicaments (Table 1).

The range of residual paste level in canal wall was maximum in CH group and minimum in PP group, as shown in Table 1. The amount of residual CH on canal walls was significantly higher than that of PP (*P*=0.001). In addition, the amount of residual CH on the canal walls was higher than that of TAP, but this difference was not significant (*P*=0.144). The amount of residual TAP on canal walls was higher than that of PP, but this difference was not significant, either (*P*=0.094). Figure 1 shows representative samples of residual CH, PP and TAP on root canal walls.

**Figure 2:**
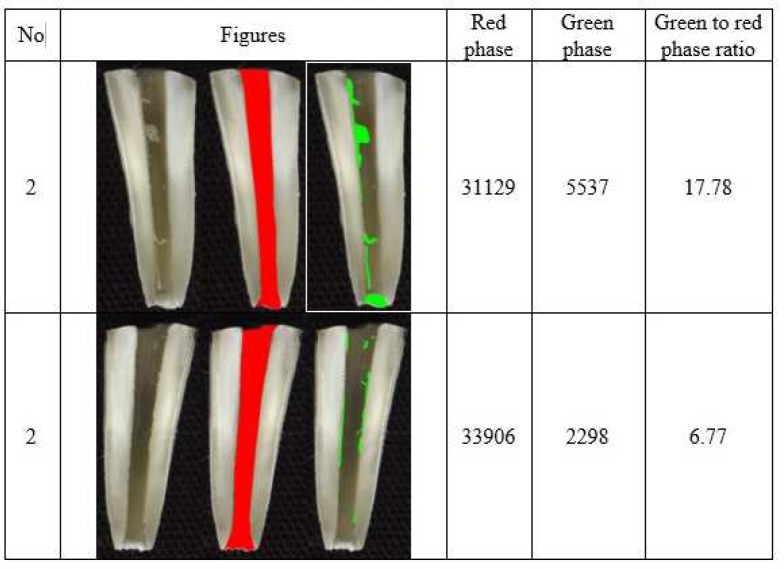
Example of propolis paste residual in canal (*Red Phase*: Total area of root canal; *Green Phase*: Area of the medicament remaining within the canal)

## Discussion

In this study, the residual levels of CH, PP and TAP on root canal walls in natural teeth with open apices were experimentally examined. In this situation the use of teeth with natural open apices is mostly similar to the natural condition.

In a systematic review and meta-analysis conducted in 2007, it was stated that CH shows limited effects in elimination of bacteria from root canals when studied by culture techniques, and the need for a more efficient antibacterial medicament to ensure complete removal of bacteria before filling the teeth was emphasized [13]. At the same time, several studies have stated several advantages for intracanal medication with CH [14, 15]. However, this compound is potentially toxic due to its high pH and can cause soft tissue damage, which may lead to chronic inflammation and cellular necrosis in clinical use [16].

Therefore, there is always need for newer and less harmful compounds in endodontic treatments with lowest irritation and highest antibacterial effect. PP is among the new materials under investigation. It is a substance with various biological properties used by honey bees to build and maintain the hive. It has anti-inflammatory, antibacterial, antioxidant, antifungal, antiviral and tissue regenerative properties [17]. In one study, the antibacterial effect of PP and TAP on *Enterococcus faecalis* was assessed and it was found that PP has more potent antibacterial effects than TAP [18].

**Figure 3:**
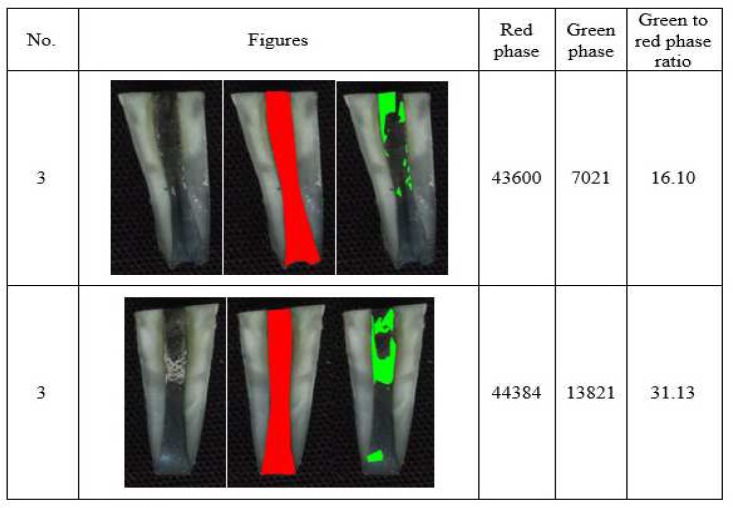
Example of triple antibiotic paste residual in canal (*Red Phase*: Total area of root canal; *Green Phase*: Area of the medicament remaining within the canal)

TAP contains ciprofloxacin, metronidazole and minocycline and has been introduced as an intracanal medicament. It is effective against the pathogens commonly found in canal, including *Enterococcus faecalis* [6, 7]. Treatment of pulp necrosis in immature teeth with an open apex always causes problems for the practitioner. Due to open apex in these teeth, obturation of the root canal and proper sealing is very difficult. Therefore, revascularization was suggested for increasing the root length and closing the apex in these teeth. Within this method, TAP was used for disinfection of the root canal. However, elimination of intra-canal medicament prior to other stages of revascularization is essential [10].

A similar study evaluated the effective removal of PP paste from canal walls of adult teeth, but the results were different from our study. Researchers stated that there is no significant difference between CH and PP groups in removal from root canal walls and the tested PP pastes had acceptable physical properties as an intracanal medicament [19]. In the present study, the residual value of CH and PP in tooth canal wall was significantly different and was maximum for CH and minimum for PP. This difference might be due to tooth type (closed *vs* open apex), method of evaluating the residual material, and different types of PP which were used.

The effect of some techniques on removal of CH as an intracanal medicament from root canal walls were investigated by some researchers and concluded that none of the techniques completely removed CH. However, ultrasonic and Canal Brush together with 5 mL of 2.5% sodium hypochlorite were more effective than irrigation with 5 mL of 2.5% NaOCl and there was no significant difference between 2.5% NaOCl and NaOCl with 17% EDTA [20].

The results of our study showed that when CH is used in root canals as a medicament, it is less likely to be removed by conventional filing and irrigation with sodium hypochlorite while PP is removed from root canals in a significantly higher amount. The removability of TAP from root canals was higher than that of CH and lower than that of PP, although this difference was not significant. Regarding TAP, the majority of studies have been concentrated on anti-bacterial properties. According to available information, no study has ever assessed the residual value of TAP in root canals.

## Conclusion

In terms of ability to be removed from root canal system, propolis is superior to calcium hydroxide. Since PP can be better removed from root canal walls than CH and TAP, if other characteristics of PP are confirmed, it could be a good alternative to conventional root canal medicaments.

## References

[1] Khadivi Nia Javan N, Mohajeri Baradaran L, Azimi S (2007). SEM Study of Root Canal Walls Cleanliness after Ni-Ti Rotary and Hand Instrumentation. Iran Endod J.

[2] Souza-Filho FJd, Soares AdJ, Vianna ME, Zaia AA, Ferraz CCR, Gomes BPFdA (2008). Antimicrobial effect and pH of chlorhexidine gel and calcium hydroxide alone and associated with other materials. Braz Dent J.

[3] Murad C, Fariniuk LF, Fidel S, Fidel RAS, Sassone LM (2008). Bacterial leakage in root canals filled with calcium hydroxide paste associated with different vehicles. Braz Dent J.

[4] Viuda‐Martos M, Ruiz‐Navajas Y, Fernández‐López J, Pérez‐Álvarez J (2008). Functional properties of honey, propolis, and royal jelly. J Food Sci.

[5] Adl A, Shojaee NS, Motamedifar M (2012). A Comparison between the Antimicrobial Effects of Triple Antibiotic Paste and Calcium Hydroxide Against Entrococcus Faecalis. Iran Endod J.

[6] Sato I, Ando‐Kurihara N, Kota K, Iwaku M, Hoshino E (1996). Sterilization of infected root‐canal dentine by topical application of a mixture of ciprofloxacin, metronidazole and minocycline in situ. Int Endod J.

[7] Hoshino E, Kurihara‐Ando N, Sato I, Uematsu H, Sato M, Kota K, Iwaku M (1996). In‐vitro antibacterial susceptibility of bacteria taken from infected root dentine to a mixture of ciprofloxacin, metronidazole and minocycline. Int Endod J.

[8] Thomson A, Kahler B (2010). Regenerative endodontics–biologically‐based treatment for immature permanent teeth: a case report and review of the literature. Aust Dent J.

[9] Martin G, Ricucci D, Gibbs JL, Lin LM (2013). Histological findings of revascularized/revitalized immature permanent molar with apical periodontitis using platelet-rich plasma. J Endod.

[10] Ding RY, Cheung GS-p, Chen J, Yin XZ, Wang QQ, Zhang CF (2009). Pulp revascularization of immature teeth with apical periodontitis: a clinical study. J Endod.

[11] Türkün M, Cengiz T (1997). The effects of sodium hypochlorite and calcium hydroxide on tissue dissolution and root canal cleanliness. Int Endod J.

[12] Erdemir A, Ari H, Güngüneş H, Belli S (2004). Effect of medications for root canal treatment on bonding to root canal dentin. J Endod.

[13] Schäfer E, Bössmann K (2005). Antimicrobial efficacy of chlorhexidine and two calcium hydroxide formulations against Enterococcus faecalis. J Endod.

[14] Shuping GB, Ørstavik D, Sigurdsson A, Trope M (2000). Reduction of intracanal bacteria using nickel-titanium rotary instrumentation and various medications. J Endod.

[15] Byström A, Claesson R, Sundqvist G (1985). The antibacterial effect of camphorated paramonochlorophenol, camphorated phenol and calcium hydroxide in the treatment of infected root canals. Dent Traumatol.

[16] Wadachi R, Araki K, Suda H (1998). Effect of calcium hydroxide on the dissolution of soft tissue on the root canal wall. J Endod.

[17] Özan F, Polat ZA, Er K, Özan Ü, Değer O (2007). Effect of propolis on survival of periodontal ligament cells: new storage media for avulsed teeth. J Endod.

[18] Madhubala MM, Srinivasan N, Ahamed S (2011). Comparative evaluation of propolis and triantibiotic mixture as an intracanal medicament against Enterococcus faecalis. J Endod.

[19] Victorino FR, Bramante CM, Zapata RO, Casaroto AR, Garcia RB, Moraes IGd, Hidalgo MM (2010). Removal efficiency of propolis paste dressing from the root canal. J Appl Oral Sci.

[20] Taşdemir T, Celik D, Er K, Yildirim T, Ceyhanli K, Yeşilyurt C (2011). Efficacy of several techniques for the removal of calcium hydroxide medicament from root canals. Int Endod J.

